# Parental Exposure to Carcinogens and Risk for Childhood Acute Lymphoblastic Leukemia, Colombia, 2000-2005

**Published:** 2011-08-15

**Authors:** Miguel Ángel Castro-Jiménez, Luis Carlos Orozco-Vargas

**Affiliations:** Centro de Investigaciones Epidemiológicas, Facultad de Salud, Universidad Industrial de Santander. Dr Castro-Jiménez is also affiliated with Grupo Colombiano de Estudios Alfa en Epidemiología, Salud Poblacional, Estadística Aplicada y Ciencias Aliadas, Bogotá, Colombia.; Universidad Industrial de Santander, Bucaramanga, Colombia

## Abstract

**Introduction:**

The objective of this study was to determine the risk factors for childhood acute lymphoblastic leukemia (ALL) and, in particular, the role of parental occupational exposure to carcinogenic and probably carcinogenic hydrocarbons before the child's conception.

**Methods:**

For this case-control study, cases were children younger than 15 years who were newly diagnosed with ALL between January 2000 and March 2005 at 1 of 6 Colombian hospitals. An interview with both parents of 170 children (85 cases and 85 individually matched neighborhood controls) gathered information about each child's exposures and parental demographic and occupational characteristics, medical history, health risk behaviors, and pregnancy and birth history. A job-exposure matrix was used to classify parental exposure to hydrocarbons on the basis of the main industrial activity of each workplace where parents worked before (both parents) or during the index pregnancy (mother only). Conditional odds ratios and 95% confidence intervals were calculated by period of exposure (preconception, pregnancy, and childhood).

**Results:**

The risk of childhood ALL was linked to 1) parental occupational exposure to hydrocarbons before conception, 2) parental smoking before conception, 3) maternal low socioeconomic status during pregnancy, and 4) higher maternal age (≥35 y) at the child's birth.

**Conclusion:**

These findings suggest an association between childhood ALL and parental occupational exposure to carcinogenic and probably carcinogenic hydrocarbons before conception. Outcomes depended on the parent exposed. Future research should investigate the additive or multiplicative role of other environmental sources of hydrocarbons.

## Introduction

Acute lymphoblastic leukemia (ALL) is the most common malignant disease in children younger than 15 years in Western countries (1). Several studies have suggested an association of childhood leukemia and other malignant diseases with some environmental or genetic parental exposures before the child's conception or during pregnancy (2-5). Parental occupational exposure to hydrocarbons has been linked to childhood ALL, but the evidence is inconclusive and the etiology of this disease remains largely unknown (6-11). The objective of this study was to determine the risk factors for childhood ALL and, in particular, to evaluate the role of parental occupational exposure to carcinogenic and probably carcinogenic hydrocarbons before the child's conception.

## Methods

### Study sample

We conducted a neighborhood-based, matched case-control study based on patients from 6 Colombian hospitals. Eligible cases consisted of all children younger than 15 years who were newly diagnosed with ALL between January 1, 2000, and March 30, 2005, and were identified through the review of institutional registries. All cases had histological and clinical verification. These registries had high levels of completeness and, if necessary, were periodically reviewed. The hospitals were 6 tertiary care centers in Bogotá and Bucaramanga, 2 major Colombian cities, and patients were residents of Bucaramanga, Bogotá, Tunja, and their closest municipalities (those located up to 2.5 hours away by car). Neighborhood-based controls were individually matched to cases (1 control per case) by sex and age at diagnosis (within or equal to 2 y of the case's age at diagnosis for cases younger than 3 y and within or equal to 3 y for cases aged 3-14 y). Controls were chosen from a house-to-house search; children recruited for the control group had to be healthy and living in the same neighborhood as the case. This area was expanded if the case child lived in a rural area where the chance to find his or her control was considered low (2 cases) or in a potentially dangerous neighborhood (1 case). Children who were adopted, who had been diagnosed with Down syndrome or any previous neoplastic disease, or who were living with people other than their biological parents were excluded.

We calculated sample size using a procedure for matched case-control studies described by Schlesselman (12). Exclusion criteria were observed in 13 (8.1%) initially eligible cases, and another 12 (8.2%) were not analyzed because the parents refused to participate. Overall, at least 1 parent of 135 cases and 113 matched controls participated. However, we based our analysis on information from 170 subjects (85 case-control pairs) with participation of both parents. Preliminary analysis suggested that unadjusted results with only these children were similar to results obtained by using the complete group of participants. If only 1 parent had been interviewed, reasons for nonparticipation of the other parent were inability to find him or her, unwillingness of the other parent to participate, and separation or death of the other parent.

### Timing of exposure and data collection

We collected data about 3 periods of the child's development: the last 24 months before conception (both parents), pregnancy (mother only), and childhood (from birth to ALL diagnosis for cases or equivalent age for controls) (mother only). These periods were calculated from the date of birth. A questionnaire-based face-to-face interview was used. This instrument (Appendices A and B) was developed by using structured questions based on a review of scientific literature. For some specific topics, a last open-ended question was added (eg, "Did you take any other medication during these last 24 months before that pregnancy?"). We asked parents about socioeconomic and demographic characteristics, work and medical history, smoking, consumption of alcoholic beverages and psychoactive drugs, and environmental exposures. To improve the likelihood that these questions would be answered, sections had a short heading with an introduction to the specific topic showing that such exposure could happen to anyone. In Colombia, socioeconomic status (SES) is classified from 1 (low) to 6 (high) in urban zones. For this study, the SES of parents who lived more than half of each period in low-income neighborhoods (including rural areas, SES1, and SES2) was classified as low and the SES of the other parents as medium/high. Nonmedical interviewers and field coordinators were trained in how to use the questionnaire through didactic sessions and a pilot study. They were not informed about the objectives or methods of this study. After cases were identified, parents were contacted by telephone or in person and the research was explained. Parents of controls were initially contacted at the time of the house-to-house search. All parents were interviewed in their houses, the hospital, or any other place they chose (eg, offices). The mean duration of the interviews was 80 minutes for mothers and 45 minutes for fathers.

### Measurement of occupational exposure

We used a job-exposure matrix. An occupational medicine physician who was also trained in occupational hygiene classified the parental exposure to hydrocarbons on the basis of the main industrial activity of each workplace where parents worked before (both parents) or during the index pregnancy (mother only). The physician was masked to the case or control status. We defined hydrocarbons classified as carcinogenic (group 1) and probably carcinogenic (group 2A) to humans by the International Agency for Research on Cancer as the occupational exposures of interest (13) (Appendix C). The main industrial activity of each workplace that parents reported was defined by the International Standard Industrial Classification (14). Parental occupational exposure to hydrocarbons (agents) and subgroups of hydrocarbons was defined by analyzing the primary economic activity of each company where the parents worked by using data from the National Occupational Exposure Survey (15).

### Statistical analysis

The difference between 2 proportions from paired data was tested by using the McNemar test or Stuart-Maxwell statistic. The difference between 2 means was assessed by using the paired t test; if distributions were not normally distributed, the Wilcoxon signed rank test was used. Odds ratios and their 95% confidence intervals were determined by using conditional logistic regression. The first step for variable selection was to discard the exposures with a P value of more than .25 in crude analysis. Possible risk factors reported in other studies (eg, mother’s age) were retained for an additional step independently of their P values. The assumption of linearity was assessed for each quantitative variable (eg, duration of breastfeeding) by plotting the log odds ratio against previously ordered categories of each, and nonlinear variables were categorized according to previous literature or the optimization method. The change-in-estimate method (16) was used for modeling, but the variable indicating parental occupational exposure to hydrocarbons during preconception (24 mo) was retained in all processes of model fitting. Preconception exposure to hydrocarbons was defined as 1) neither parent exposed, 2) father only exposed, 3) mother only exposed, and 4) both parents exposed. The likelihood ratio was used to test the significance of the difference between the fitted model and a reduced model. Pairs with subjects determined to be poorly fit were identified by using graphical methods explained by Hosmer and Lemeshow (17). Significance was set at P < .05. Stata SE version 9.0 (StataCorp LP, College Station, Texas) was used for analysis. Written informed consent was obtained from the parents of all participating children. This study was approved by the Industrial University of Santander ethical committee, the Javeriana University ethical committee, and the institutional review boards of the participating hospitals. 

## Results

### Matching aspects

The median difference in age between cases and controls was 1.12 years (interquartile range, 0.52-1.91 y). For 7 cases, we were unable to find a control of the same sex, but comparative analyses with and without these pairs suggested that their exclusion did not affect the risks found in this study. These 7 pairs were included in the analysis. The other variables matched.

### Crude analysis

The distribution of parental socioeconomic and demographic characteristics between cases and controls was similar for each period (Table 1). Parental occupational exposures to hydrocarbons of interest at home and in other workplaces were combined. The prevalence of parental occupational exposure to most of these hydrocarbons was higher among cases than among controls. Unadjusted significant associations were found between childhood ALL and preconception maternal exposures to mineral oil, aliphatic, and aromatic subgroups, and to the following specific agents: trichloroethylene, benzene, epichlorohydrin, ethylene oxide, and diesel engine exhaust (Table 2a). Preconception paternal exposure to the mineral oil subgroup and trichloroethylene agents also showed significant effects. Maternal exposures to any specific hydrocarbon during the index pregnancy did not significantly affect the crude risk of ALL (Table 2b).

The storage of chemical products in 1 or more of the mother's houses and the exposure to 1 or more types of pesticides during pregnancy, but not other environmental exposures, were significantly associated with an increased risk of childhood ALL (Table 3).

In most of the conditions investigated, the prevalence of maternal and paternal diseases before the child's conception was lower than 10% and did not reach significance. These results were similar to the prevalence of maternal diseases during the index pregnancy. The crude association of some parental diseases with childhood ALL is shown in Table 3. Paternal smoking before conception and passive maternal smoking during pregnancy were significantly associated with childhood ALL. Parental consumption of alcohol did not show any significant association with childhood ALL. No significant associations were found between childhood ALL and parental exposure to psychoactive drugs (Table 3).

The mean breastfeeding duration was higher in the case group (19.7 mo) than in the control group (15.2 mo). Controls were significantly more likely than cases to have been breastfed for 6 to 11 months (Table 4).

### Multivariable analysis (fitted model)

After the model-fitting process, parental occupational exposure to 1 or more hydrocarbons classified as carcinogenic or probably carcinogenic before the child's conception was associated with an increased risk of childhood ALL. The risk was also higher with preconception parental exposure to smoking, elevated maternal age at index birth, and low maternal SES during index pregnancy (Table 5). The likelihood ratio test showed that the fitted model was better than 2 reduced models (the first one containing only intercept and the second one containing intercept plus explanatory variable). The multivariable analyses also showed that the parental occupational exposure to some of the more prevalent hydrocarbons classified as carcinogenic or probably carcinogenic to humans is related to a higher risk of childhood ALL after adjusting for nonoccupational confounders described in Table 5 (Table 6).

## Discussion

Our findings showed a significantly higher risk of ALL among children whose parents were occupationally exposed to carcinogenic and probably carcinogenic hydrocarbons before the child's conception. These results also suggest that maternal exposure to these chemicals is more important than paternal exposure. The exposure to hydrocarbons during and before pregnancy has been linked to an increased risk of childhood ALL and brain tumors (6,7,18). Other studies have not found this association (8-10), but this lack of consistency may be partially explained by methodologic differences such as the statistical procedures used in modeling, the range of variables covered, or the use of proxies to obtain data. Our study's sample size was low; the resulting confidence intervals were wide and should be interpreted with caution.

The "2-hit" theory of carcinogenesis states that a minimum of 2 mutagenic events is necessary to facilitate cancer development and that the first mutagenic hit may be inherited by the offspring from a germ line mutation in 1 of the parental genes (19). The role of the germ cell mutations has been debated. Rinaldi et al suggest that childhood ALL is of single-cell origin and that germ cell mutations are unlikely to play a major role in the pathogenesis of ALL (20). In our results, which were similar to those of Shu et al (6), parental occupational exposure to hydrocarbons and to active or passive smoking, also a source of carcinogenic hydrocarbons, may contribute to childhood ALL as a first hit before conception. Our analysis strongly suggests that paternal smoking, alone or in combination with maternal smoking, before conception is an important risk factor for childhood ALL. This result is similar to that published by Ji et al (21) and Chang et al (22). Although the association between childhood ALL and maternal active smoking is unclear, we found a link to maternal passive smoking during gestation. Data on parental smoking were gathered by using a section of the questionnaire based on a complete history of parental smoking, and recall bias is an unlikely explanation of this finding.

The distribution of socioeconomic and demographic characteristics between cases and neighborhood controls was expected to be remarkably similar, but advanced maternal age at the child's birth was significantly associated with an increased risk for childhood ALL; this finding agrees with other studies (23-25). The selection of controls was based on where the case child was living at the time of diagnosis, and the distribution of socioeconomic and demographic variables were considered unknown for periods before and during pregnancy (these variables could change over time). In fact, our analysis also shows that childhood ALL may be related to maternal low SES during pregnancy. Other studies have suggested that this factor is inversely associated, or not associated, with childhood ALL (26,27).

Most studies have shown that breastfeeding is significantly associated with a change in the risk for childhood ALL (28-31). Our data suggest that the duration of breastfeeding, analyzed as a quantitative variable (in months), has a clear nonlinear pattern with the U-shaped relationship; its highest protective effect was observed for children who had been exposed for 6 to 11 months. However, this factor did not achieve significance and was not retained in the fitted model. The parental history of diseases before and during pregnancy and their exposures to medications, psychoactive drugs, and alcohol were not associated with an increased risk of ALL in their children. Other studies have shown controversial or contradictory results (32,33).

One of the most important characteristics of a case-control study is that the controls should be chosen from the same population that gives rise to cases, and its major limitation is that it is highly susceptible to selection bias (34). We consider selection bias unlikely because 1) we found that the proportion of births by cesarean was similar between cases and controls, and 2) the prevalence of smoking in Bucaramanga among parents of controls was similar to that from a cross-sectional study carried out in the general population of Bucaramanga, the same group that presumably gave rise to the group of cases in this study (27.7% and 26.3% for men and 11.1% and 10.5% for women, respectively) (35). However, even in a biased control population, some exposures will have a similar proportion, and information related to other variables was not compared. Furthermore, it is necessary to take into account that controls were selected at the time of the case diagnosis but, during the time between the diagnosis and the interview, some eligible controls could leave the neighborhood, resulting in potential selection bias.

Matching on neighborhood is considered a convenient substitute for population-based sampling of controls if the source population cannot be enumerated (34). However, a case-control study using a questionnaire is prone to recall bias because the disease has already occurred when information is obtained and the exposure often took place a long time ago (36); it is also possible that the parents of the children with ALL were interviewed after becoming educated about possible risk factors for the disease (36).

On the other hand, the use of a nonspecific job exposure matrix may have resulted in incorrect classification of the exposure to hydrocarbons. The high correlation among some hydrocarbons (data not shown) may be a product of a combination of a true mix of these agents and the matrix's possible low ability to discriminate among them.

In conclusion, this study has several limitations and its results may be prone to bias. However, these findings support the hypothesis that parental occupational exposure to some hydrocarbons before conception may be related to an increased risk of childhood ALL. Other possible sources of carcinogenic hydrocarbons (parental smoking and maternal exposure to pollution, indirectly measured by SES during pregnancy) were retained in the fitted model. New studies are needed to explore the difference in risk of childhood ALL according to which parent had been exposed to hydrocarbons.

## Figures and Tables

**Table 1:** Crude Association Between Childhood ALL and Parental Demographic Characteristics, Colombia, 2000-2005

**Characteristic**	No. of Cases (n = 85)^a^	No. of Controls (n = 85)^a^	OR (95% CI)
**Maternal**			
**Age at child's birth, y**			
<35	71	75	1 [Reference]
≥35	14	10	1.57 (0.60-4.05)
**SES during 24 months before conception^b^**			
High	25	27	1 [Reference]
Low	60	58	1.16 (0.54-2.52)
**SES during index pregnancy^b^**			
Medium or high	25	31	1 [Reference]
Low	60	54	1.66 (0.73-3.80)
**Area of residence during 24 months before conception**			
Urban only	77	80	1 [Reference]
Any rural	8	5	0.62 (0.20-1.91)
**Area of residence during index pregnancy**			
Urban only	78	77	1 [Reference]
Any rural	7	8	1.14 (0.41-3.15)
**Education at child's birth (in years completed)**			
0-5	32	23	1 [Reference]
6-11	42	56	0.49 (0.24-1.04)
≥12	11	6	1.41 (0.39-5.10)
**Paternal**			
**Age at child's birth, y**			
<35	56	55	1 [Reference]
≥35	29	30	0.93 (0.46-1.90)
**SES during 24 months before conception^b^**			
Medium or high	28	27	1 [Reference]
Low	57	58	0.91 (0.40-2.08)
**Area of residence during 24 months before conception**			
Urban only	74	78	1 [Reference]
Any rural	11	7	0.55 (0.19-1.66)
**Education at child's birth (in years completed)**			
0-5	29	27	1 [Reference]
6-11	44	45	0.90 (0.46-1.79)
≥12	12	13	0.85 (0.34-2.18)

Abbreviations: ALL, acute lymphoblastic leukemia; OR, odds ratio; CI, confidence interval; SES, socioeconomic status.

a Cases were children aged 0-14 y who were newly diagnosed with ALL between January 2000 and March 2005. Neighborhood-based controls were matched to cases by sex and age at diagnosis.

b In Colombia, SES is classified from 1 (low) to 6 (high) in urban zones. Parents who lived more than half of the index pregnancy in low-income neighborhoods (including rural area, SES1, and SES2) were classified as having low SES.

**Table 2a:** Crude Association Between Childhood ALL and Parental Occupational Exposure to Carcinogenic and Probably Carcinogenic Hydrocarbons Before Child's Conception, Colombia, 2000-2005

**Hydrocarbon Subgroup or Agent^a^**	No. of Cases (n = 85)^b^	No. of Controls (n = 85)^b^	OR (95% CI)	*P* Value
**Maternal**				
Mineral oils	30	15	3.14 (1.34-7.35)	.01
Aliphatic	31	16	3.50 (1.41-8.67)	.01
Amides	14	9	1.83 (0.67-4.95)	.23
Aromatics	31	16	3.50 (1.41-8.67)	.01
1,3-Butadiene	10	5	2.66 (0.70-10.05)	.15
Trichloroethylene	31	16	3.50 (1.41-8.67)	.01
Acrylamide	14	9	1.83 (0.67-4.95)	.23
Benzene	30	16	3.00 (1.27-7.05)	.01
Benzyl chloride	19	10	2.28 (0.94-5.55)	.07
Epichlorohydrin	13	5	3.66 (1.02-13.14)	.046
Ethylene oxide	28	15	2.85 (1.20-6.75)	.02
Coal-tar pitches	4	3	1.50 (0.25-8.97)	.66
Diesel engine exhaust	31	15	3.66 (1.48-9.04)	.01
ortho-Toluidine	21	12	2.12 (0.91-4.92)	.01
**Paternal**				
Mineral oils	51	36	2.15 (1.12-4.16)	.02
Aliphatic	53	45	1.57 (0.80-3.07)	.19
Amides	18	11	1.87 (0.79-4.42)	.15
Aromatics	53	45	1.57 (0.80-3.07)	.19
1,3-Butadiene	24	13	2.10 (0.99-4.46)	.05
Trichloroethylene	52	37	2.15 (1.12-4.16)	.02
Acrylamide	18	11	1.87 (0.79-4.42)	.15
Benzene	34	25	1.60 (0.84-3.05)	.15
Benzyl chloride	14	10	1.44 (0.62-3.38)	.39
Epichlorohydrin	28	22	1.35 (0.72-2.53)	.34
Ethylene oxide	32	32	1.00 (0.53-1.89)	>.99
Coal-tar pitches	18	12	1.66 (0.72-3.80)	.27
Diesel engine exhaust	49	45	1.23 (0.65-2.34)	.52
ortho-Toluidine	23	20	1.21 (0.59-2.46)	.59

Abbreviations: ALL, acute lymphoblastic leukemia; OR, odds ratio; CI, confidence interval.

a Children whose parents were not occupationally exposed to these hydrocarbons were considered nonexposed and were the reference group.

b Cases were children aged 0-14 y who were newly diagnosed with ALL between January 2000 and March 2005. Neighborhood-based controls were matched to cases by sex and age at diagnosis.

**Table 2b:** Crude Association Between Childhood ALL and Maternal Occupational Exposure to Carcinogenic and Probably Carcinogenic Hydrocarbons During Index Pregnancy, Colombia, 2000-2005

**Hydrocarbon Subgroup or Agent^a^**	No. of Cases (n = 85)^b^	No. of Controls (n = 85)^b^	OR (95% CI)	*P* Value
Mineral oils	22	15	1.77 (0.79-4.02)	.17
Aliphatic	24	15	2.12 (0.92-4.92)	.08
Amides	8	9	0.87 (0.32-2.41)	.80
Aromatics	24	15	2.12 (0.92-4.92)	.08
1,3-Butadiene	7	4	2.00 (0.50-7.99)	.33
Trichloroethylene	24	15	2.12 (0.92-4.92)	.08
Acrylamide	8	9	0.87 (0.32-2.41)	.80
Benzene	23	15	1.88 (0.84-4.24)	.12
Benzyl chloride	16	8	2.33 (0.90-6.07)	.08
Epichlorohydrin	10	6	1.80 (0.60-5.37)	.29
Ethylene oxide	21	15	1.66 (0.73-3.80)	.28
Coal-tar pitches	3	4	0.75 (0.16-3.35)	.71
Diesel engine exhaust	22	14	1.88 (0.84-4.24)	.12
ortho-Toluidine	16	10	1.75 (0.73-4.17)	.21

Abbreviations: ALL, acute lymphoblastic leukemia; OR, odds ratio; CI, confidence interval.

a Children whose mothers were not occupationally exposed to these hydrocarbons were considered nonexposed and were the reference group.

b Cases were children aged 0-14 y who were newly diagnosed with ALL between January 2000 and March 2005. Neighborhood-based controls were matched to cases by sex and age at diagnosis.

**Table 3:** Crude Odds Ratios for Childhood ALL, by Parental Clinical Conditions, Risk Behaviors, and Environmental Exposures Before and During Index Pregnancy, Colombia, 2000-2005^a^

Characteristic	Preconception (24 Months, Inclusive)^b^		Index Pregnancy^b^

	Mother, OR (95% CI)	Father, OR (95% CI)	Mother, OR (95% CI)
**Diseases and x-ray exposure**			
Urinary tract infection	2.33 (0.60-9.02)	0.50 (0.05-5.51)	1.36 (0.63-2.97)
Any infection	2.20 (0.76-6.33)	1.50 (0.53-4.21)	1.38 (0.67-2.82)
Hypertension^c^	3.00 (0.61-14.86)	4.00 (0.45-35.79)	1.57 (0.61-4.05)
X-ray	1.33 (0.56-3.16)	1.25 (0.58-2.67)	2.00 (0.18-22.06)
**Behaviors**			
Active smoking	1.18 (0.53-2.64)	1.93 (1.06-3.54)	1.16 (0.39-3.47)
Passive smoking	1.57 (0.91-2.71)	1.50 (0.76-2.95)	2.00 (1.07-3.71)
Alcohol use	0.87 (0.43-1.79)	3.00 (0.60-14.87)	0.95 (0.51-1.78)
Use of psychoactive drugs	NE	2.00 (0.37-10.91)	NC
Passive exposure to psychoactive drugs^d^	0.75 (0.17-3.35)	0.84 (0.38-1.89)	1.66 (0.40-6.97)
**Environmental exposures in 1 or more houses (to a distance <200 m)**			
Agriculture area	1.85 (0.74-4.65)	1.00 (0.40-2.52)	1.83 (0.68-4.96)
Power tower	1.50 (0.42-5.32)	0.30 (0.08-1.09)	1.00 (0.29-3.45)
Power transformers	1.00 (0.46-2.15)	1.05 (0.55-2.01)	1.06 (0.54-2.10)
Gas station	0.90 (0.39-2.14)	1.22 (0.51-2.96)	1.16 (0.39-3.47)
Use or storage of any chemical product	1.78 (0.93-3.44)	0.85 (0.40-1.85)	2.20 (1.04-4.64)
Petroleum	2.18 (1.07-4.45)	1.62 (0.67-3.92)	2.12 (0.92-4.92)
Pesticides	1.33 (0.56-3.16)	1.40 (0.44-4.41)	2.80 (1.01-7.77)

Abbreviations: ALL, acute lymphoblastic leukemia; OR, odds ratio; CI, confidence interval; NE, none exposed; NC, not calculated (only 1 case exposed).

a Based on 85 case-control pairs. Cases were children aged 0-14 y who were newly diagnosed with ALL between January 2000 and March 2005. Neighborhood-based controls were matched to cases by sex and age at diagnosis.

b Children who were not exposed to these conditions were considered nonexposed and were the reference group.

c This variable may indicate chronic hypertension, preeclampsia, or both during index pregnancy.

d Defined as the inhalation of smoke from a psychoactive drug (eg, marijuana) by a person who was not the direct user.

**Table 4:** Crude Odds Ratios for Childhood ALL, by Child Characteristics, Colombia, 2000-2005

**Characteristic**	No. of Cases (n = 85)^a^	No. of Controls (n = 85)^a^	OR (95% CI)
**Cesarean delivery**			
No	61	61	1 [Reference]
Yes	24	24	1.00 (0.52-1.92)
**Gestational age, wk^b^**			
<37	9	13	1 [Reference]
37-41	69	69	1.45 (0.56-3.80)
≥42	7	3	3.16 (0.66-15.20)
**Birth weight, g^b^**			
<2,500	10	10	1 [Reference]
2,500-3,999	63	68	0.84 (0.33-2.20)
≥4,000	12	5	2.41 (0.61-9.49)
**Birth height, cm^b^**			
<47	9	11	1 [Reference]
47-54	66	68	1.33 (0.53-3.32)
≥55	10	5	2.61 (0.66-10.34)
**Breastfeeding duration, mo^c^**			
0 or ≥18	48	37	1 [Reference]
1-5	17	15	0.83 (0.38-1.84)
6-11	10	22	0.36 (0.15-0.87)
12-17	10	11	0.70 (0.25-1.95)
**Any infection**			
No	58	62	1 [Reference]
Yes	27	23	1.22 (0.66-2.28)
**Exposure to x-ray**			
No	52	49	1 [Reference]
Yes	31	36	0.78 (0.42-1.45)
**SES classification^d^**			
Medium or high	26	30	1 [Reference]
Low	59	55	1.66 (0.61-4.59)
**Residence zone**			
Urban only	75	76	1 [Reference]
Any rural	10	9	0.87 (0.32-2.41)

Abbreviations: ALL, acute lymphoblastic leukemia; OR, odds ratio; CI, confidence interval; SES, socioeconomic status.

a Cases were children aged 0-14 y who were newly diagnosed with ALL between January 2000 and March 2005. Neighborhood-based controls were matched to cases by sex and age at diagnosis.

b Mothers who did not remember this information exactly were asked if doctors or nurses had talked to them about this variable (higher, lower, or similar than the expected value).

c Children showed a similar crude risk in 0 (5 cases, 3 controls) or ≥18 m (34 cases, 43 controls); to obtain more statistical power, their data were combined.

d In Colombia, SES is classified from 1 (low) to 6 (high) in urban zones. Children who lived more than half of their life in low-income neighborhoods (including rural area, SES1, and SES2) were classified as having low SES.

**Table 5:** Fitted Model of Multivariate Odds Ratios for Risk for Childhood ALL, by Parental Characteristics, Colombia, 2000-2005^a^

**Characteristic**	OR (95% CI)
**Parental occupational exposure to hydrocarbons^b^ during 24 months before conception**	
Neither parent	1 [Reference]
Father only	1.66 (0.64-4.28)
Mother only	6.33 (1.41-28.31)
Both parents	13.47 (3.31-54.71)
**Parental active smoking during 24 months before conception**	
Neither parent	1 [Reference]
One or both parents	2.63 (1.24-5.56)
**Maternal SES during index pregnancy^c^**	
Medium or high	1 [Reference]
Low	3.53 (1.22-10.21)
**Maternal age at child's birth, y**	
<35	1 [Reference]
≥35	3.72 (1.13-12.29)

Abbreviations: ALL, acute lymphoblastic leukemia; OR, odds ratio; CI, confidence interval; SES, socioeconomic status.

a Based on 85 case-control pairs. Cases were children aged 0-14 y who were newly diagnosed with ALL between January 2000 and March 2005. Neighborhood-based controls were matched to cases by sex and age at diagnosis.

b At least 1 hydrocarbon classified as carcinogenic or probably carcinogenic to humans (Appendix C).

c In Colombia, SES is classified from 1 (low) to 6 (high) in urban zones. Mothers who lived more than half of the index pregnancy in low-income neighborhoods (including rural area, SES1, and SES2) were classified as having low SES.

**Table 6:** Multivariate Odds Ratios for Association Between Childhood ALL and Parental Occupational Exposure to Hydrocarbons Classified as Carcinogenic or Probably Carcinogenic to Humans, Colombia, 2000-2005^a^

Hydrocarbon Subgroup or Agent	Exposure^b^		

	Father Only, OR (95% CI)	Mother Only, OR (95% CI)	Both Parents, OR (95% CI)
Mineral oils	2.92 (1.16-7.36)	6.68 (1.59-28.08)	13.68 (3.58-52.22)
Aliphatic	1.66 (0.64-4.28)	6.33 (1.41-28.31)	13.47 (3.31-54.71)
Amides	2.20 (0.81-5.92)	3.25 (0.75-13.96)	5.36 (0.93-30.91)
Aromatics	1.66 (0.64-4.28)	6.33 (1.41-28.31)	13.47 (3.31-54.71)
1,3-Butadiene	4.18 (1.47-11.88)	11.67 (1.74-78.05)	2.05 (0.12-29.27)
Trichloroethylene	2.76 (1.09-7.06)	7.41 (1.66-33.07)	17.56 (4.12-74.81)
Acrylamide	2.20 (0.81-5.92)	3.25 (0.75-13.96)	5.36 (0.93-30.91)
Benzene	1.74 (0.72-4.17)	5.50 (1.38-21.92)	11.65 (2.98-45.59)
Benzyl chloride	1.37 (0.41-4.56)	3.03 (0.95-9.65)	7.66 (1.20-48.69)
Epichlorohydrin	2.00 (0.86-4.64)	6.59 (1.05-41.32)	11.56 (1.67-79.80)
Ethylene oxide	0.78 (0.31-1.96)	3.85 (1.05-14.09)	7.98 (2.10-30.28)
Coal-tar pitches	2.66 (0.98-7.25)	2.24 (0.23-21.38)	2.19 (0.10-44.37)
Diesel engine exhaust	1.29 (0.52-3.16)	6.70 (1.51-29.69)	11.26 (2.80-45.24)
ortho-Toluidine	1.47 (0.58-3.73)	3.26 (0.96-11.04)	6.44 (1.43-28.94)

Abbreviations: ALL, acute lymphoblastic leukemia; OR, odds ratio; CI, confidence interval.

a Multivariate odds for matched case-control pairs (n = 85) after adjustment for maternal age at child's birth, parental preconception smoking status, and maternal socioeconomic status during index pregnancy. Cases were children aged 0-14 y who were newly diagnosed with ALL between January 2000 and March 2005. Neighborhood-based controls were matched to cases by sex and age at diagnosis.

b Children whose parents were not occupationally exposed to these hydrocarbons were considered nonexposed and were the reference group.

## Appendices

### Appendix A: Questionnaire About Mother

**Questionnaire to Assess Child's Exposures and Mother's Demographic and Occupational Characteristics, Medical History, Health Risk Behaviors, and Pregnancy and Birth History, Colombia, 2000-2005** (DOC 1.1Mb, Spanish Document). This file is available for download as a Microsoft Word document.

**Description: **Microsoft Word is a word processing program used to create and edit text documents. Text in Word documents can be easily modified or copied for use in other applications.

**File extensions: **.doc, .rtf

**Viewing: **If you do not already have Word, you can download Word Viewer for free.

### Appendix B: Questionnaire About Father

**Questionnaire to Assess Father's Demographic and Occupational Characteristics, Medical History, and Health Risk Behaviors, Colombia, 2000-2005** (DOC 491k, Spanish Document). This file is available for download as a Microsoft Word document.

**Description: **Microsoft Word is a word processing program used to create and edit text documents. Text in Word documents can be easily modified or copied for use in other applications.

**File extensions: **.doc, .rtf

**Viewing: **If you do not already have Word, you can download Word Viewer for free.

### Appendix C: Hydrocarbons Classified as Carcinogenic or Probably Carcinogenic to Humans

**Table:**

**Group 1: Carcinogenic to humans**

Benzene [71-43-2] (Vol. 29, Suppl. 7; 1987)
Benzidine [92-87-5] (Vol. 29, Suppl. 7; 1987)
Bis(chloromethyl)ether [542-88-1] and chloromethyl methyl ether [107-30-2] (technical-grade) (Vol. 4, Suppl. 7; 1987)
Ethylene oxide [75-21-8] (Vol. 60; 1994)
2-Naphthylamine [91-59-8] (Vol. 4, Suppl. 7; 1987)
2,3,7,8-Tetrachlorodibenzo-para-dioxin [1746-01-6] (Vol. 69; 1997)
Coal-tar pitches [65996-93-2] (Vol. 35, Suppl. 7; 1987)
Coal-tars [8007-45-2] (Vol. 35, Suppl. 7; 1987)
Mineral oils, untreated and mildly treated (Vol. 33, Suppl. 7; 1987)
Shale oils [68308-34-9] (Vol. 35, Suppl. 7; 1987)

**Group 2A: Probably carcinogenic to humans**

Acrylamide [79-06-1] (Vol. 60; 1994)
Benz[*a*]anthracene [56-55-3] (Vol. 32, Suppl. 7; 1987)
Benzo[*a*]pyrene [50-32-8] (Vol. 32, Suppl. 7; 1987)
1,3-Butadiene [106-99-0] (Vol. 71; 1999)
a-Chlorinated toluenes (benzal chloride [98-87-3], benzotrichloride [98-07-7], benzyl chloride [100-44-7]), and benzoyl chloride [98-88-4] (combined exposures) (Vol. 29, Suppl. 7 and Vol. 71; 1999)
4-Chloro-ortho-toluidine [95-69-2] (Vol. 77; 2000)
Epichlorohydrin [106-89-8] (Vol. 11, Suppl. 7 and Vol. 71; 1999)
Ethylene dibromide [106-93-4] (Vol. 15, Suppl. 7 and Vol. 71; 1999)
4,4?-Methylene bis(2-chloroaniline) (MOCA) [101-14-4] (Vol. 57; 1993)
N-Nitrosodimethylamine [62-75-9] (Vol. 17, Suppl. 7; 1987)
Styrene-7,8-oxide [96-09-3] (Vol. 60; 1994)
Tetrachloroethylene [127-18-4] (Vol. 63; 1995)
ortho-Toluidine [95-53-4] (Vol. 77; 2000)
Trichloroethylene [79-01-6] (Vol. 63; 1995)
1,2,3-Trichloropropane [96-18-4] (Vol. 63; 1995)
Vinyl bromide [593-60-2] (Vol. 39, Suppl. 7 and Vol. 71; 1999)
Diesel engine exhaust (Vol. 46; 1989)
Petroleum refining (occupational exposures in) (Vol. 45; 1989)

Source: International Agency for Research on Cancer (13).
